# Supplementary material for: Triterpenoids from the Leaves of Cyclocarya paliurus and Their Glucose Uptake Activity in 3T3-L1 Adipocytes
Source: Molecules. 2023 Apr 7;28(8):3294. doi: 10.3390/molecules28083294 (PMC10145478; doi:10.3390/molecules28083294)
Supplement: Supplementary file 1 [file molecules-28-03294-s001.zip › molecules-2292292-supplementary.pdf]

# Triterpenoids from the Leaves of Cyclocarya paliurus and Their Glucose Uptake Activity in 3T3-L1 Adipocytes

**Xiaoqin Liang 1, Shengping Deng 1, Yan Huang 1, Liwei Pan 1, Yanling Chang 1, Ping Hou 1, Chenyang Ren 1, Weifeng Xu 1, Ruiyun Yang 1, Kanyuan Li 1, Jun Li 1,* and Ruijie He 2,***

1 State Key Laboratory for Chemistry and Molecular Engineering of Medicinal Resources/Key Laboratory for Chemistry and Molecular Engineering of Medicinal Resources (Ministry of Education of China), Collaborative Innovation Center for Guangxi Ethnic Medicine, School of Chemistry and Pharmaceutical Sciences, Guangxi Normal University, Guilin 541004, China; yang_rui_yun@163.com (R.Y.)
2 Guangxi Key Laboratory of Plant Functional Phytochemicals and Sustainable Utilization, Guangxi Institute of Botany, Guangxi Zhuang Autonomous Region and Chinese Academy of Sciences, Guilin 541006, China
* Correspondence: lijun9593@gxnu.edu.cn (J.L.); heruijie927@163.com (R.H.)

**Figures of Contents**

Figure S1. HRESIMS spectrum of compound **1** .................................................. 4
Figure S2. $^{1}$H NMR spectrum of compound **1** (600 MHz, pyridine-$d_5$) ............... 4
Figure S3. $^{13}$C NMR spectrum of compound **1** (150 MHz, pyridine-$d_5$) .............. 5
Figure S4. DEPT spectrum of compound **1** in pyridine-$d_5$ .................................. 5
Figure S5. HSQC spectrum of compound **1** in pyridine-$d_5$ .................................. 6
Figure S6. $^{1}$H-$^{1}$H COSY spectrum of compound **1** in pyridine-$d_5$ ........................ 6
Figure S7. HMBC spectrum of compound **1** in pyridine-$d_5$ ................................. 7
Figure S8. ROESY spectrum of compound **1** in pyridine-$d_5$ ................................ 7
Figure S9. HRESIMS spectrum of compound **2** .................................................. 8
Figure S10. $^{1}$H NMR spectrum of compound **2** (600 MHz, pyridine-$d_5$) ............. 8
Figure S11. $^{13}$C NMR spectrum of compound **2** (150 MHz, pyridine-$d_5$) ............ 9
Figure S12. DEPT spectrum of compound **2** in pyridine-$d_5$ ................................ 9
Figure S13. HSQC spectrum of compound **2** in pyridine-$d_5$ .............................. 10
Figure S14. $^{1}$H-$^{1}$H COSY spectrum of compound **2** in pyridine-$d_5$ .................... 10
Figure S15. HMBC spectrum of compound **2** in pyridine-$d_5$ ............................. 11
Figure S16. ROESY spectrum of compound **2** in pyridine-$d_5$ ............................ 11
Figure S17. HRESIMS spectrum of compound **3** .............................................. 12
Figure S18. $^{1}$H NMR spectrum of compound **3** (600 MHz, pyridine-$d_5$) ........... 12
Figure S19. $^{13}$C NMR spectrum of compound **3** (150MHz, pyridine-$d_5$) ........... 13
Figure S20. DEPT spectrum of compound **3** in pyridine-$d_5$ .............................. 13
Figure S21. HSQC spectrum of compound **3** in pyridine-$d_5$ .............................. 14
Figure S22. $^{1}$H-$^{1}$H COSY spectrum of compound **3** in pyridine-$d_5$ .................... 14
Figure S23. HMBC spectrum of compound **3** in pyridine-$d_5$ ............................. 15
Figure S25. HRESIMS spectrum of compound **4** .............................................. 16
Figure S26. $^{1}$H NMR spectrum of compound **4** (600 MHz, pyridine-$d_5$) ........... 16
Figure S27. $^{13}$C NMR spectrum of compound **4** (150MHz, pyridine-$d_5$) ........... 17
Figure S28. DEPT spectrum of compound **4** in pyridine-$d_5$ .............................. 17
Figure S29. HSQC spectrum of compound **4** in pyridine-$d_5$ .............................. 18
Figure S30. $^{1}$H-$^{1}$H COSY spectrum of compound **4** in pyridine-$d_5$ .................... 18
Figure S31. HMBC spectrum of compound **4** in pyridine-$d_5$ ............................. 19
Figure S32. ROESY spectrum of compound **4** in pyridine-$d_5$ ............................ 19
Figure S33. The HPLC spectrum of the standard of L-arabinose ........................ 20
Figure S34. The HPLC spectrum of the standard of D-glucopyranose ............... 20

Figure S35. The HPLC spectrum of the standard of D-quinovose ...................... 20
Figure S36. The HPLC spectrum of the compound **1** of L-arabinose ................. 21
Figure S37. The HPLC spectrum of the compound **2** and **3** of D-glucopyranose and L-arabinose ........................................................................................ 21
Figure S38. The HPLC spectrum of the compound **4** of L-arabinose and D-quinovose ........................................................................................... 21

# Figures of Contents

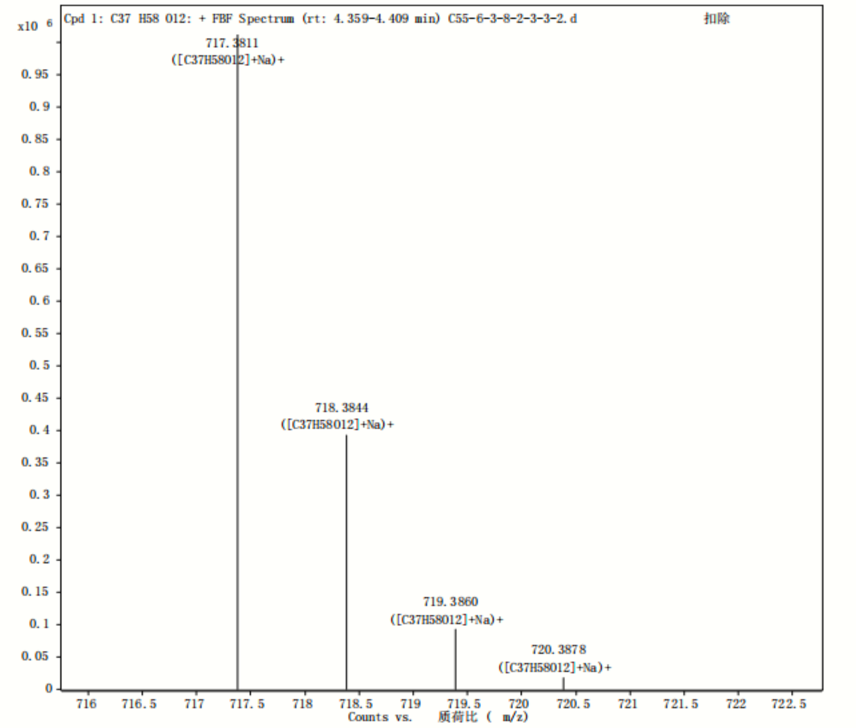


**Figure S1**. HRESIMS spectrum of compound **1**

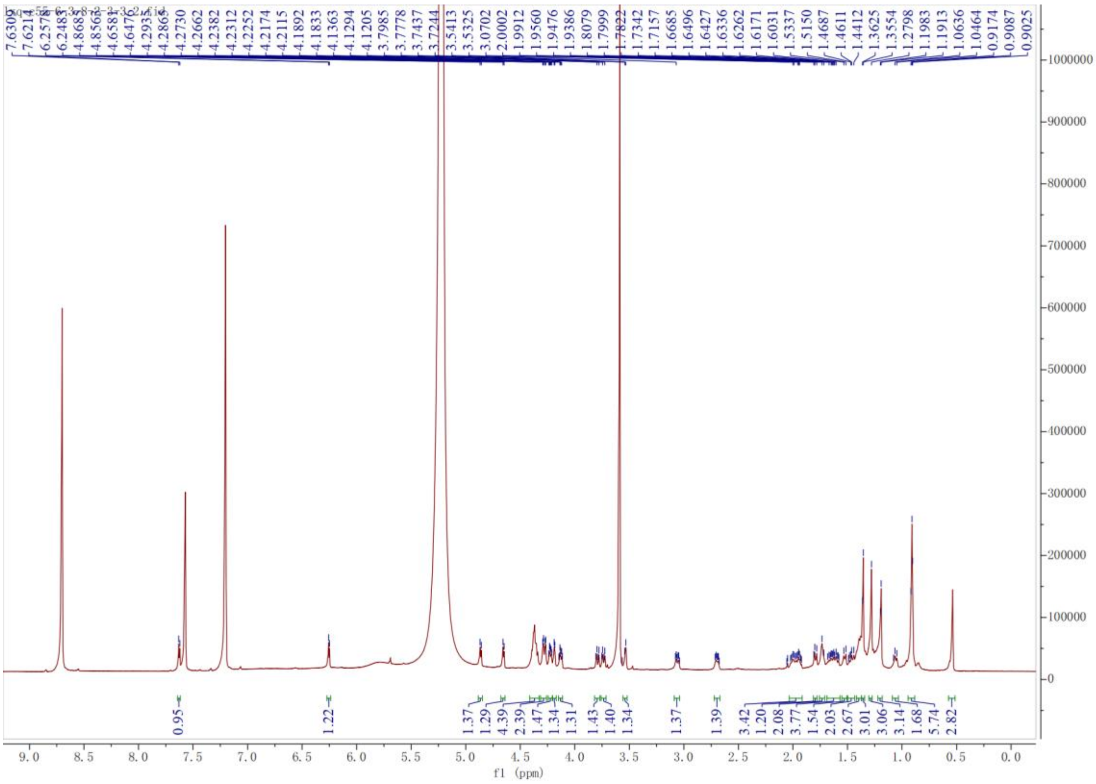


**Figure S2**. $^{1}$H NMR spectrum of compound **1** (600 MHz, pyridine-$d_5$)

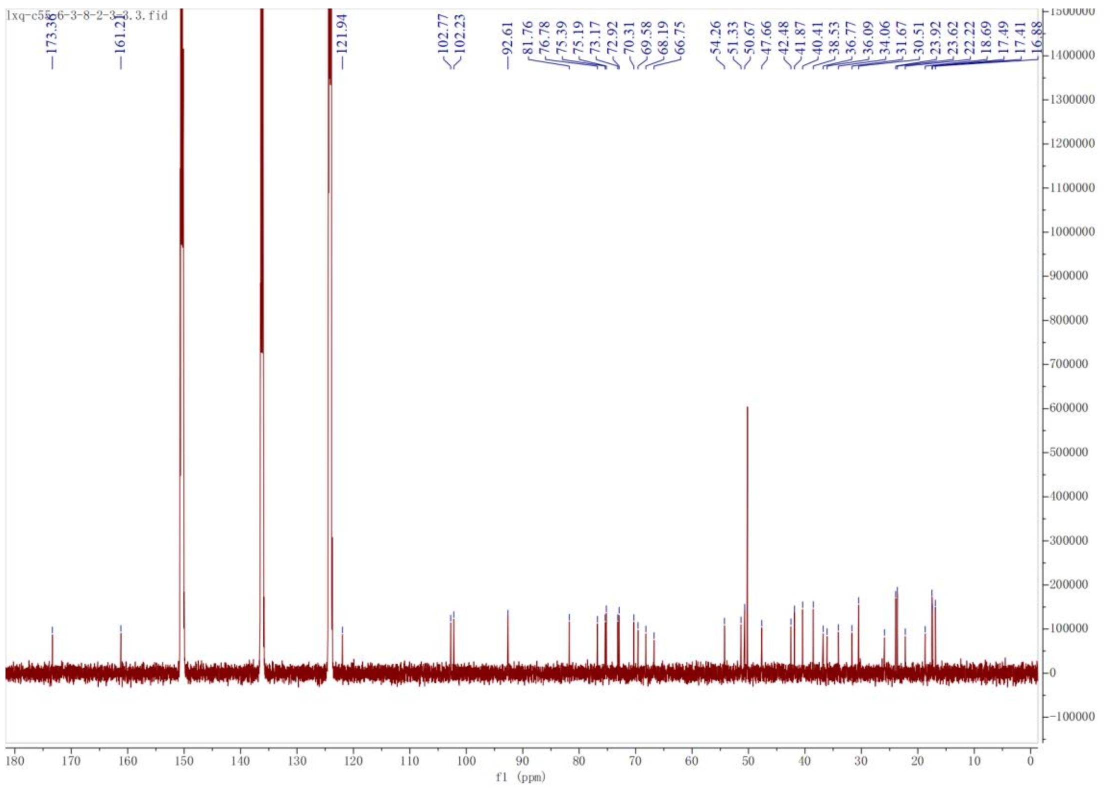


**Figure S3**. $^{13}$C NMR spectrum of compound **1** (150 MHz, pyridine-$d_5$)

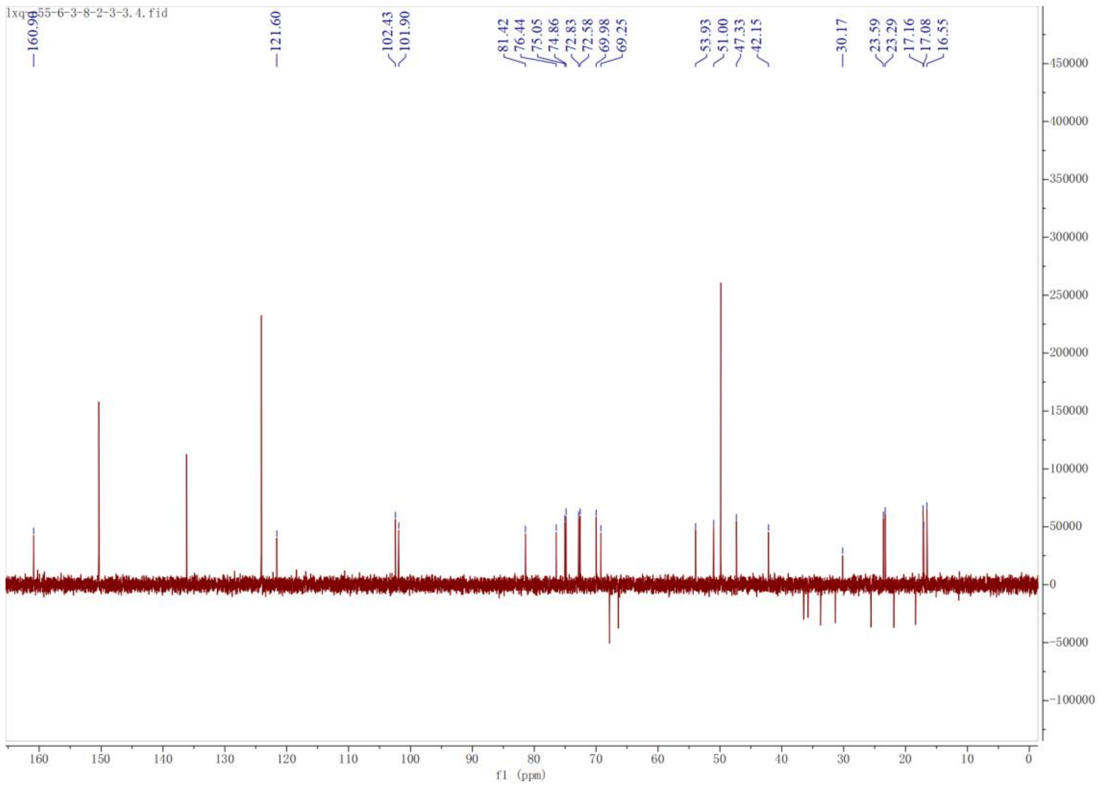


**Figure S4**. DEPT spectrum of compound **1** in pyridine-$d_5$

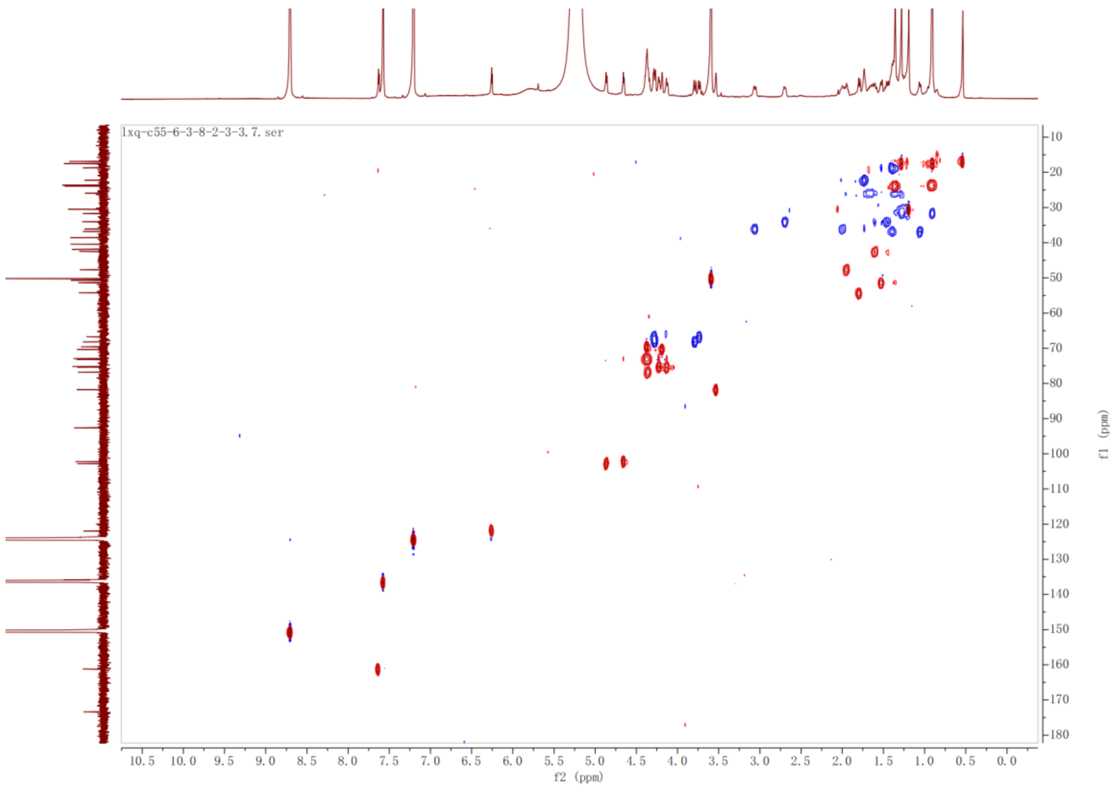


**Figure S5**. HSQC spectrum of compound **1** in pyridine-*d*5

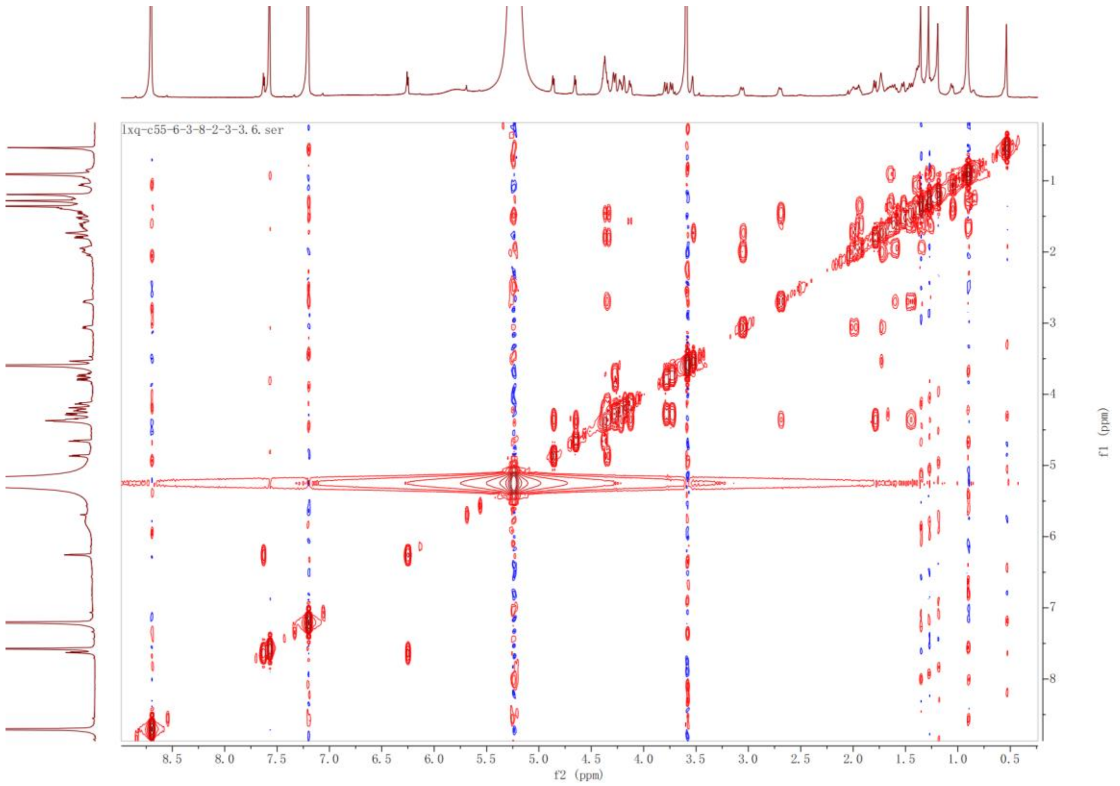


**Figure S6**. $^1$H-$^1$H COSY spectrum of compound **1** in pyridine-*d*5

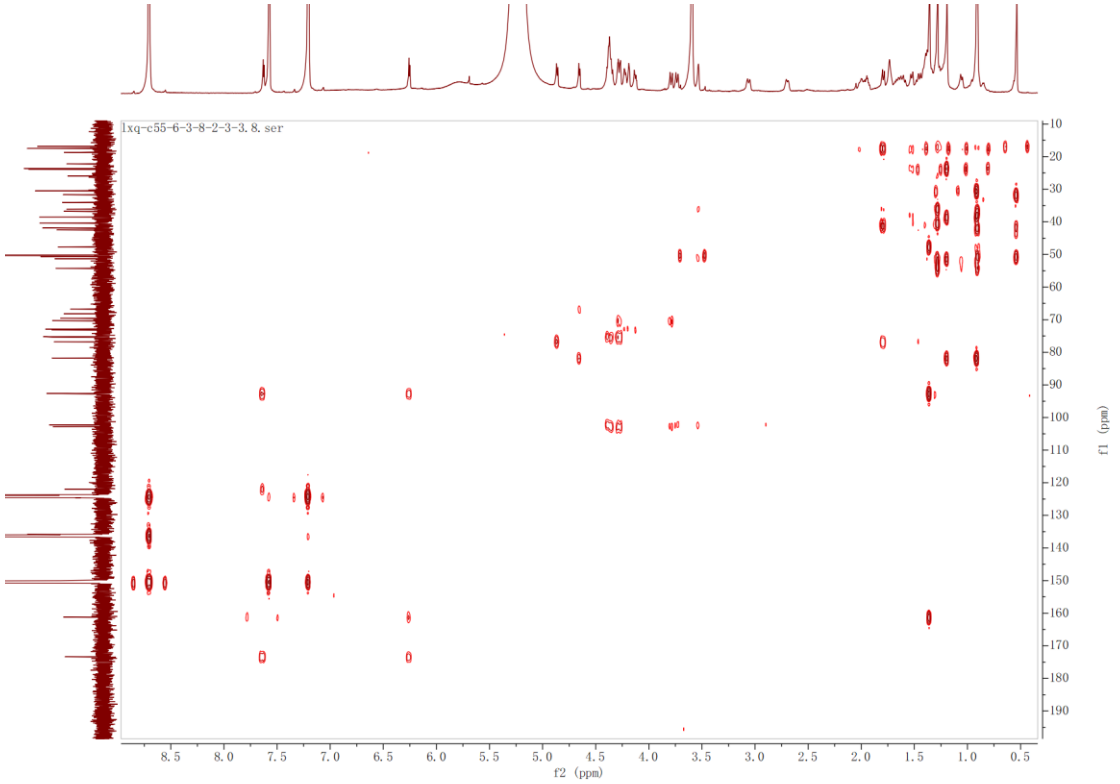


**Figure S7**. HMBC spectrum of compound **1** in pyridine-$d_5$

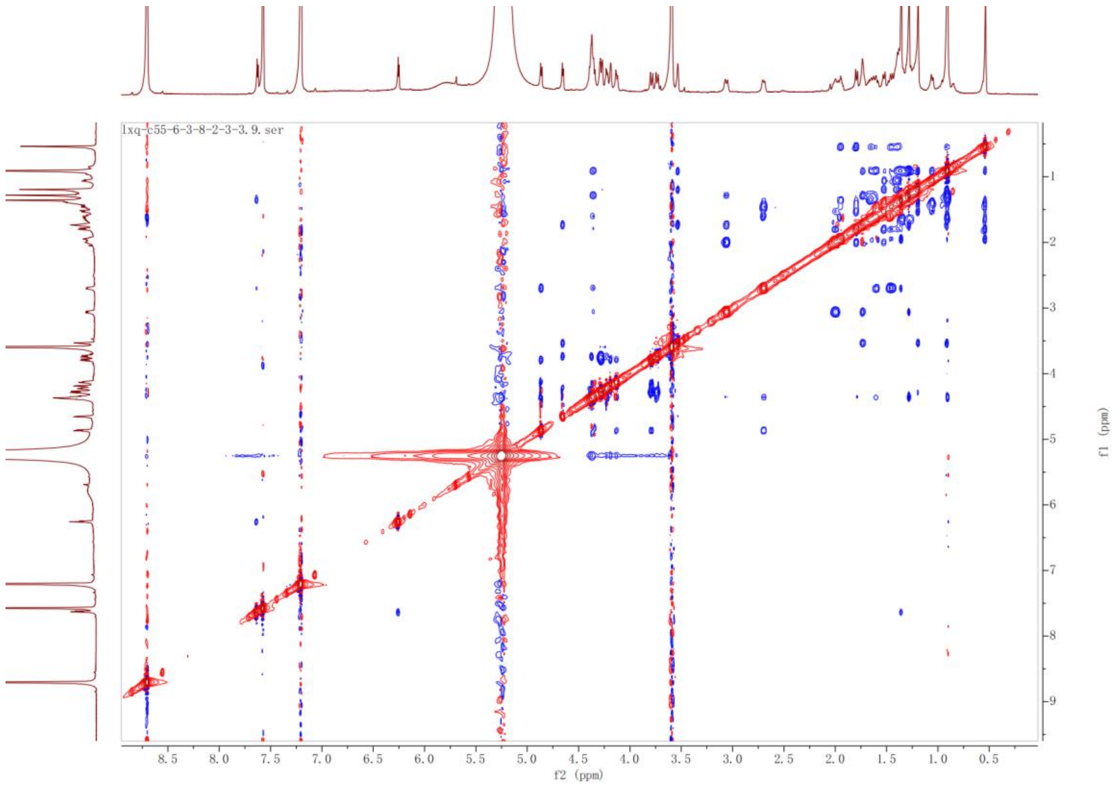


**Figure S8**. ROESY spectrum of compound **1** in pyridine-$d_5$

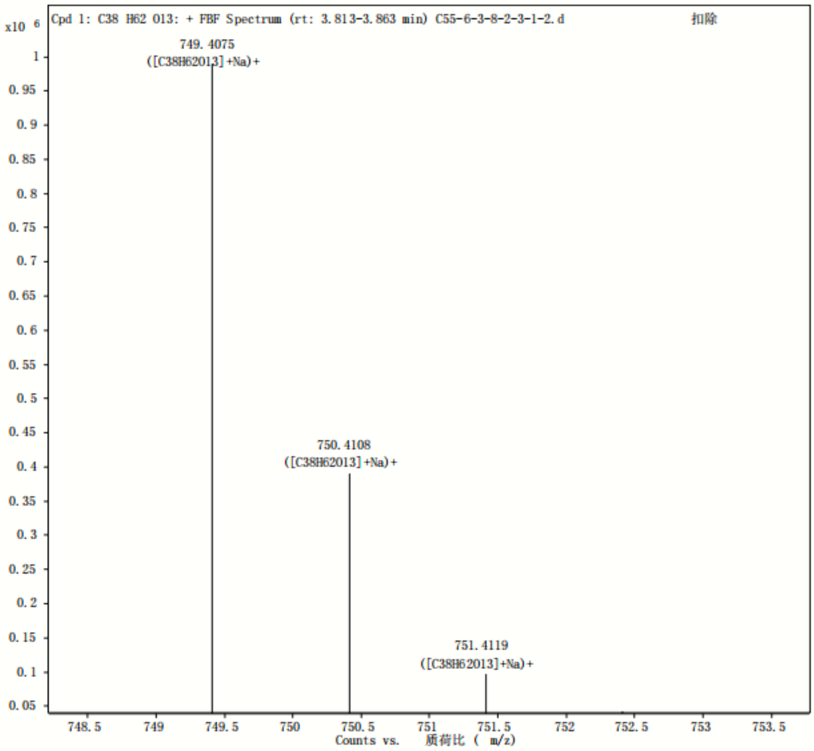


**Figure S9**. HRESIMS spectrum of compound **2**

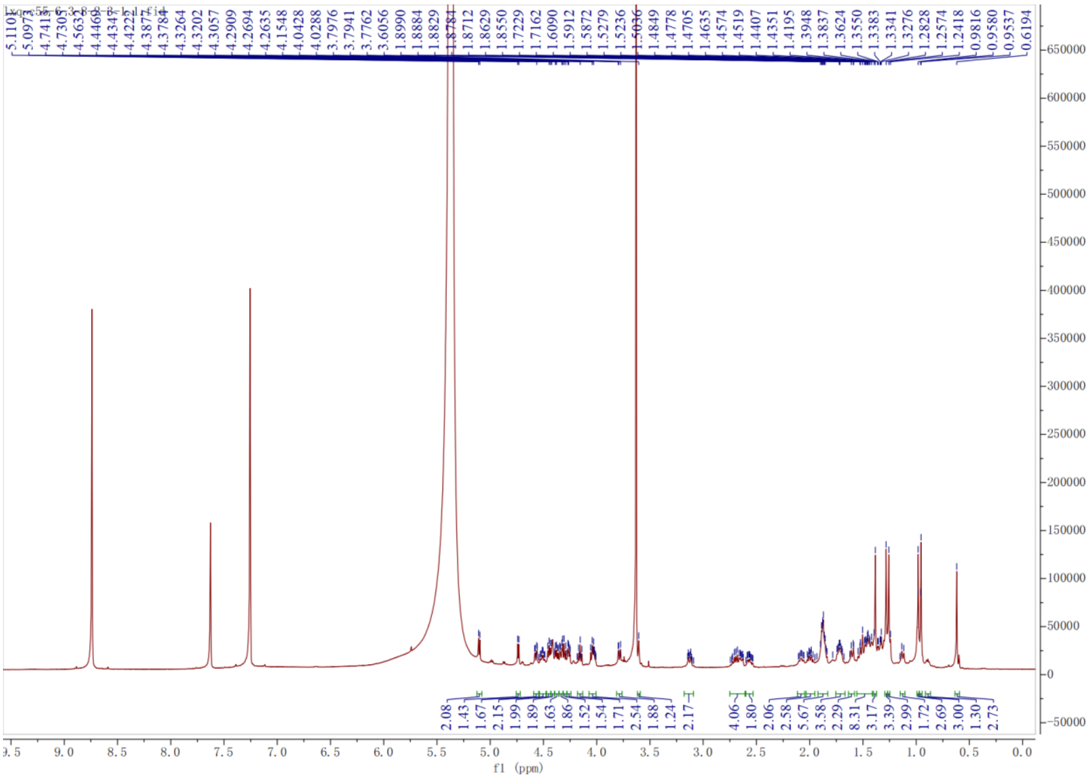


**Figure S10**. $^{1}$H NMR spectrum of compound **2** (600 MHz, pyridine-$d_5$)

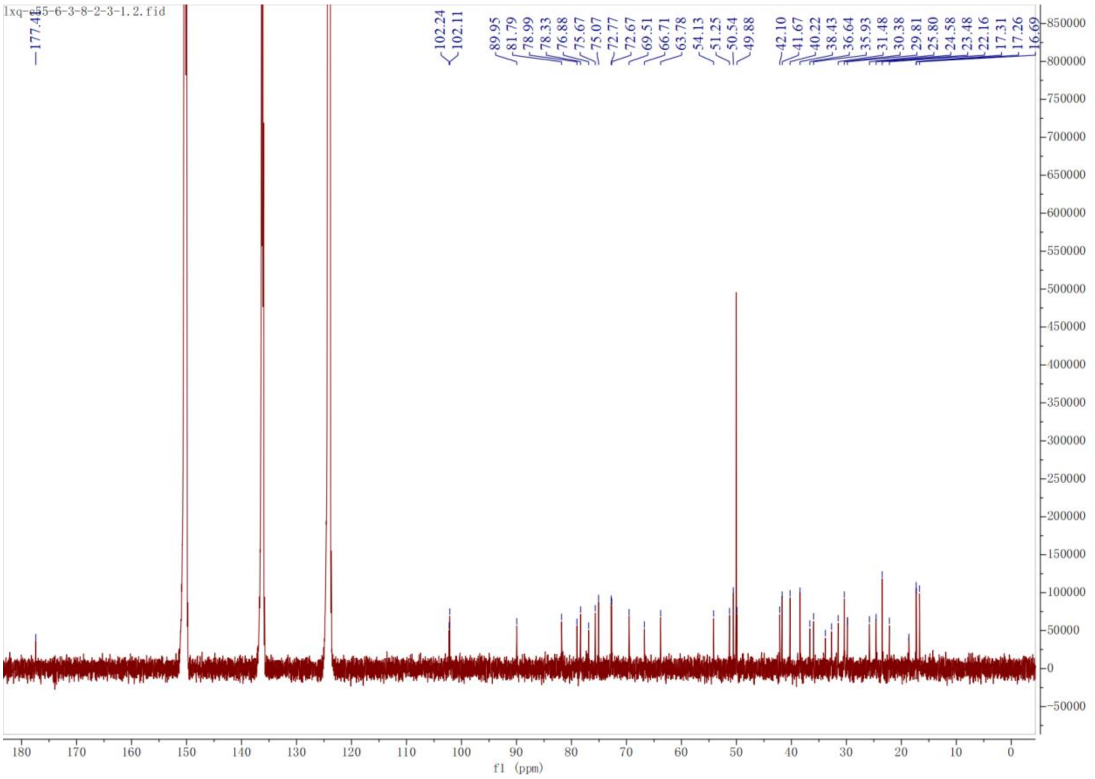

**Figure S11**. $^{13}$C NMR spectrum of compound **2** (150 MHz, pyridine-$d_5$)

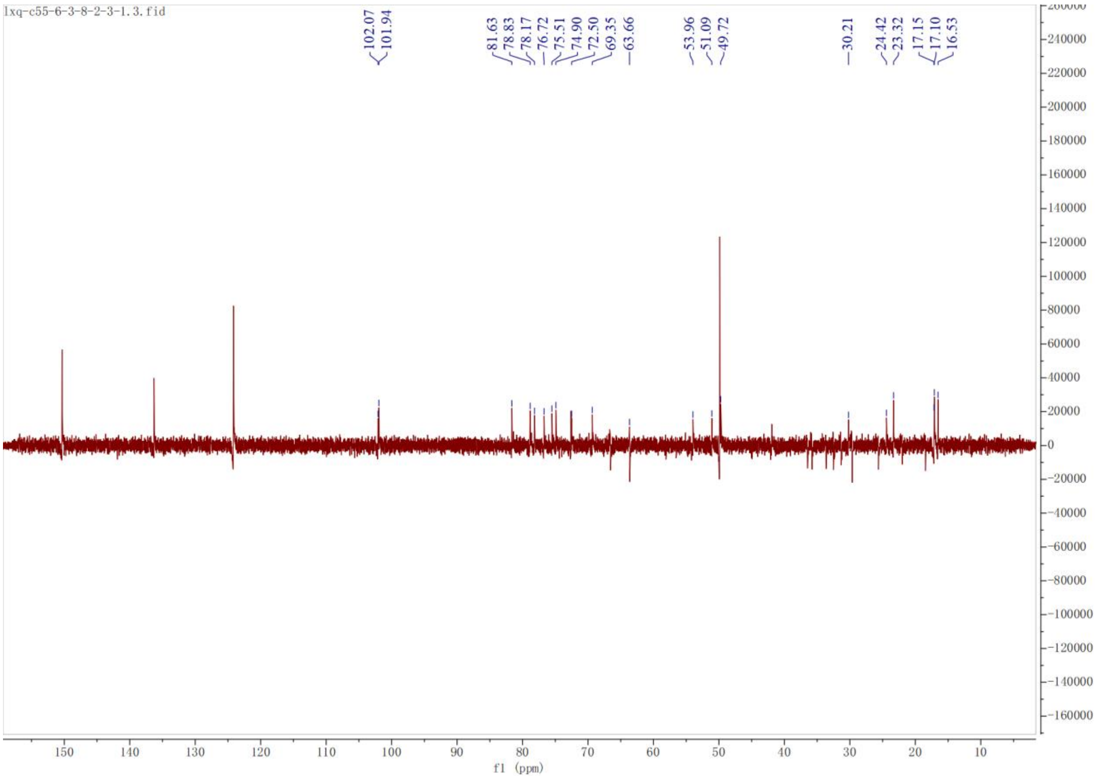

**Figure S12**. DEPT spectrum of compound **2** in pyridine-$d_5$

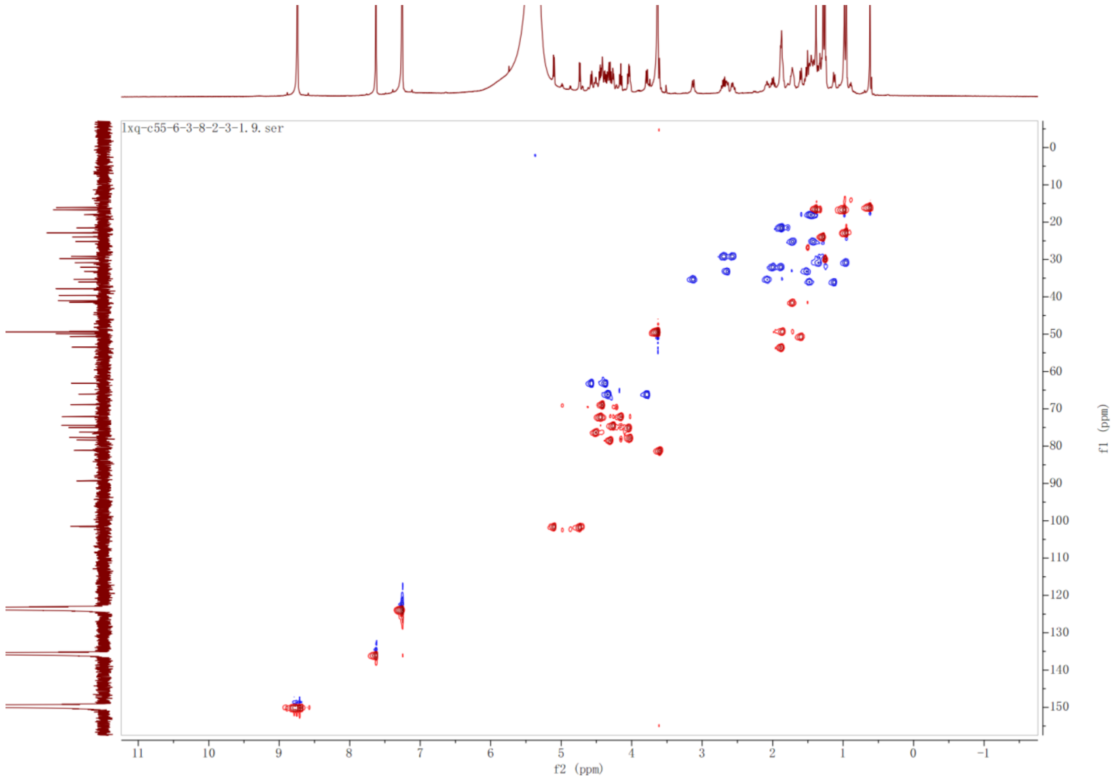


**Figure S13**. HSQC spectrum of compound **2** in pyridine-$d_5$

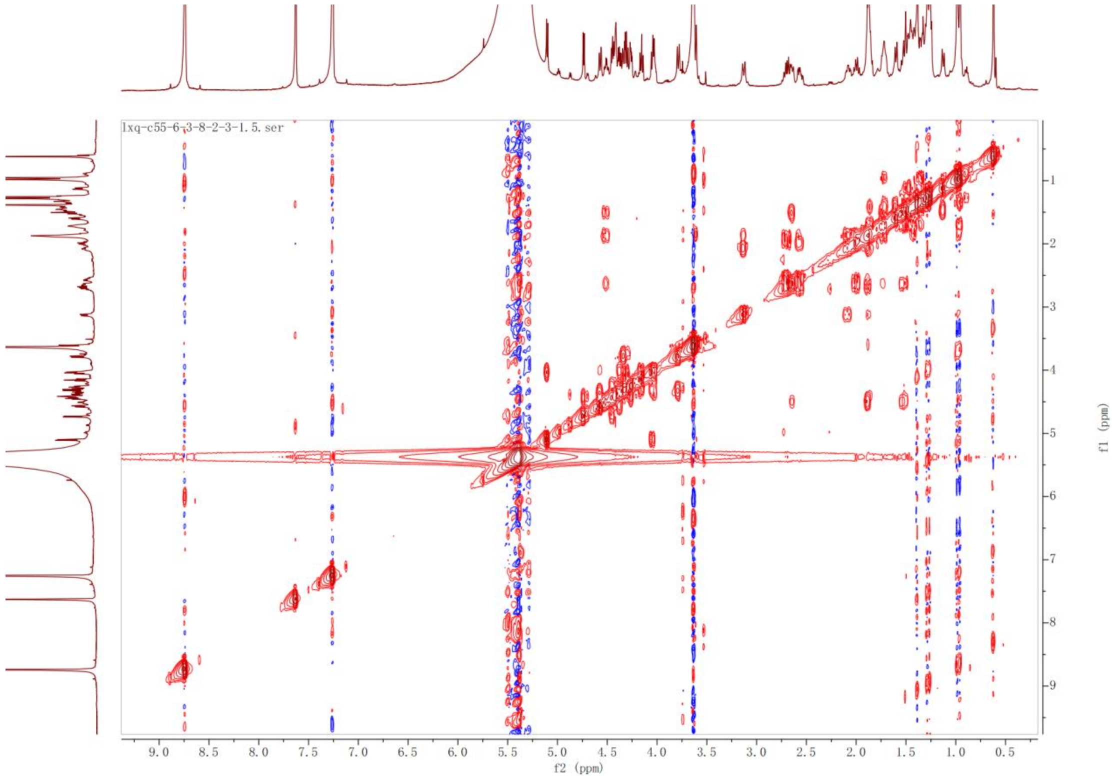


**Figure S14**. $^1$H-$^1$H COSY spectrum of compound **2** in pyridine-$d_5$

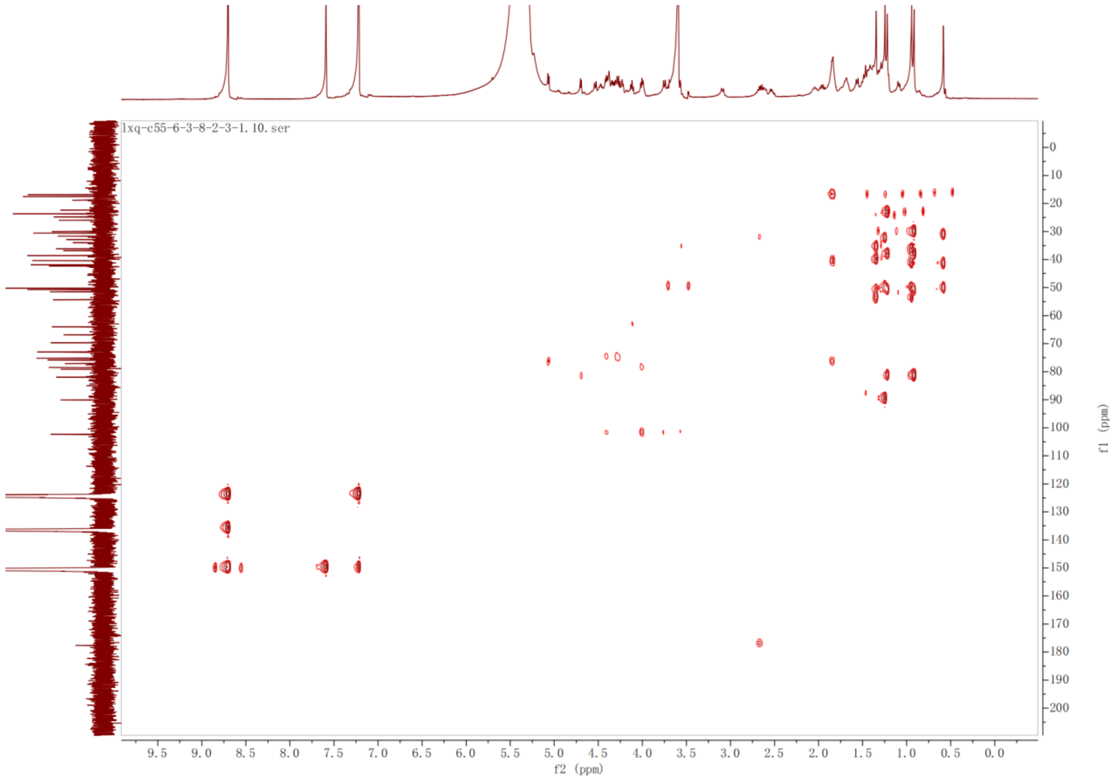


**Figure S15**. HMBC spectrum of compound **2** in pyridine-$d_5$

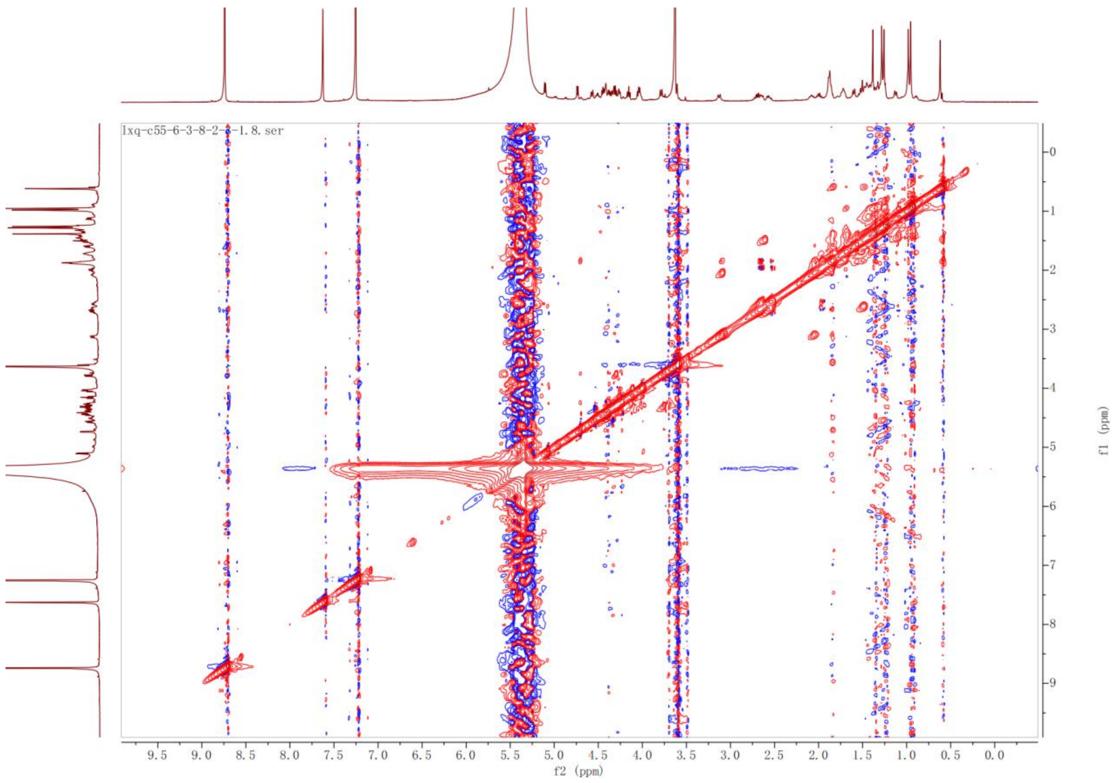


**Figure S16**. ROESY spectrum of compound **2** in pyridine-$d_5$

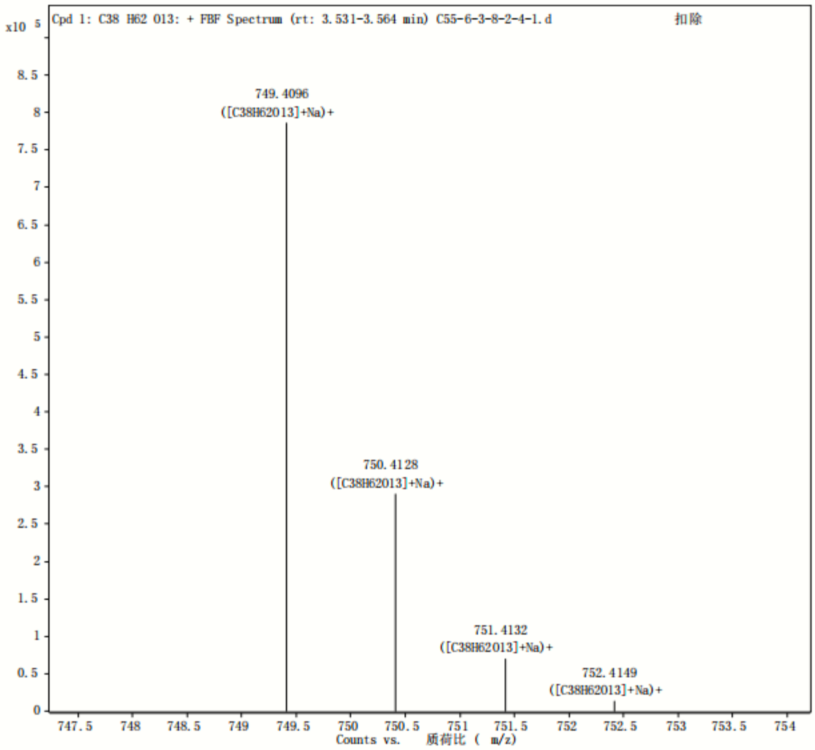


**Figure S17**. HRESIMS spectrum of compound **3**

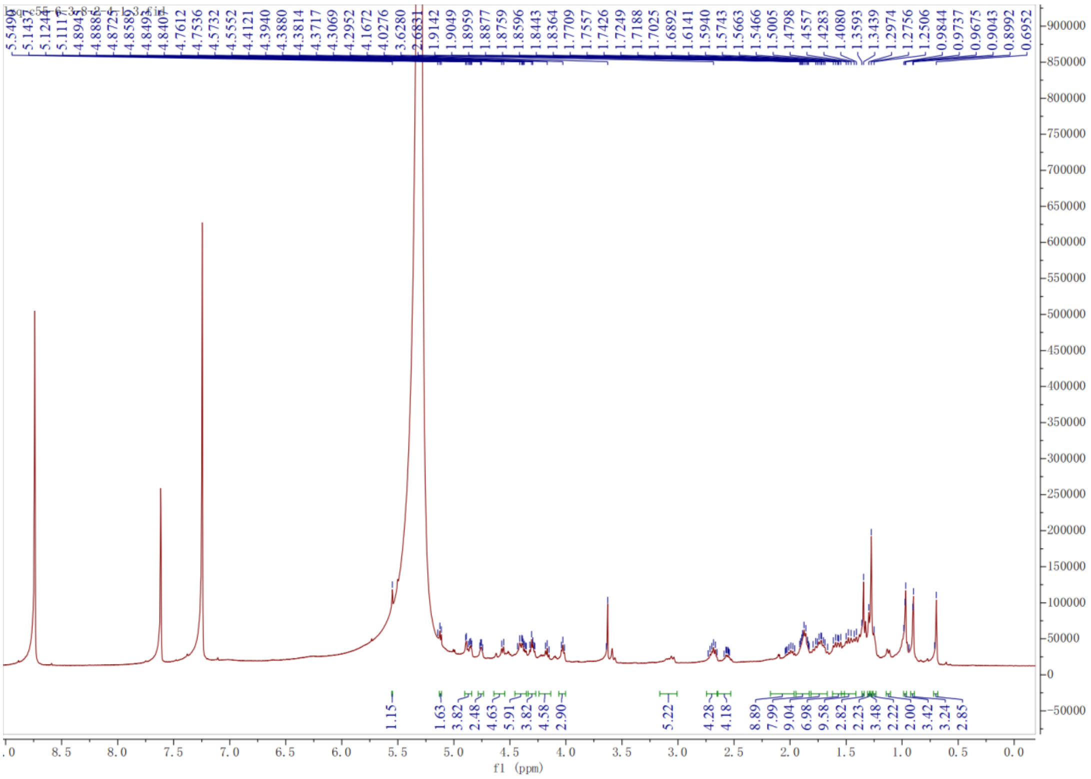


**Figure S18**. $^{1}$H NMR spectrum of compound **3** (600 MHz, pyridine-$d_5$)

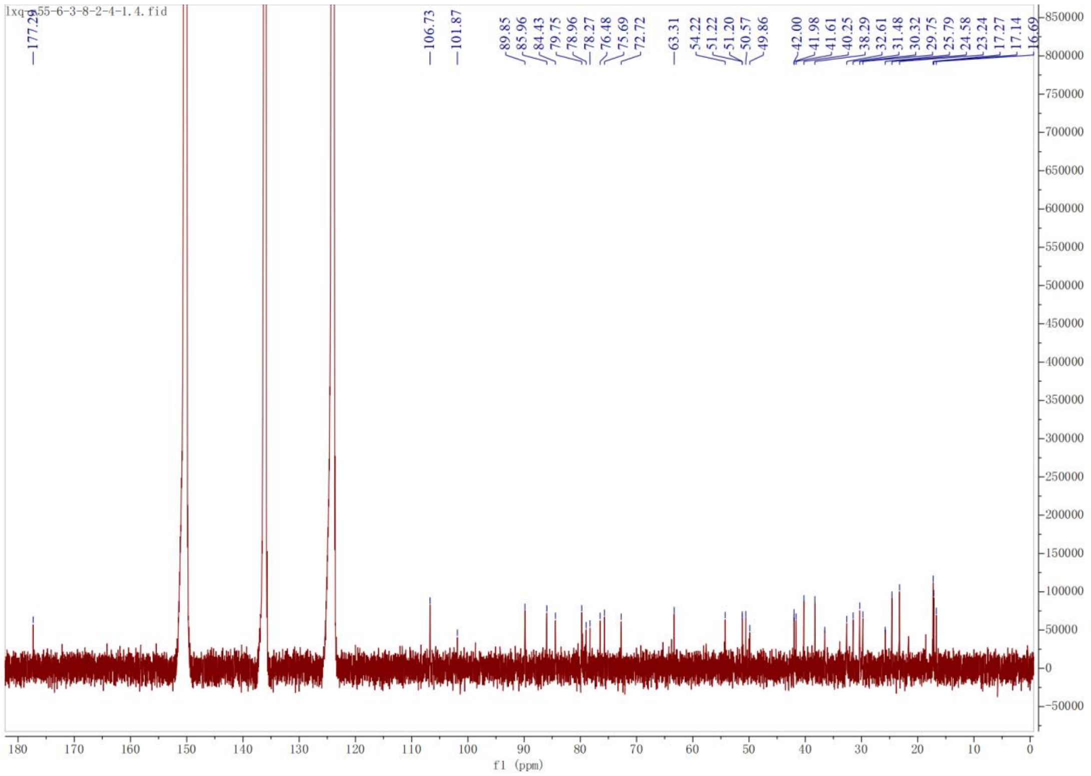


**Figure S19**. $^{13}C$ NMR spectrum of compound **3** (150MHz, pyridine-$d_5$)

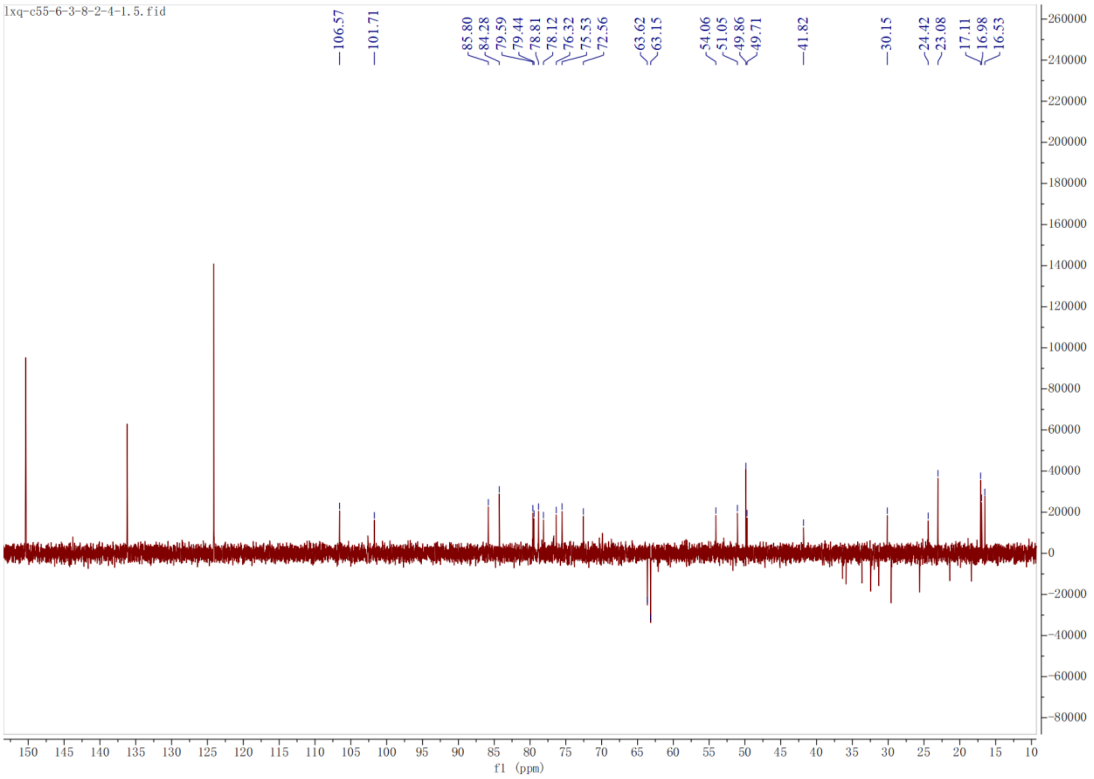


**Figure S20**. DEPT spectrum of compound **3** in pyridine-$d_5$

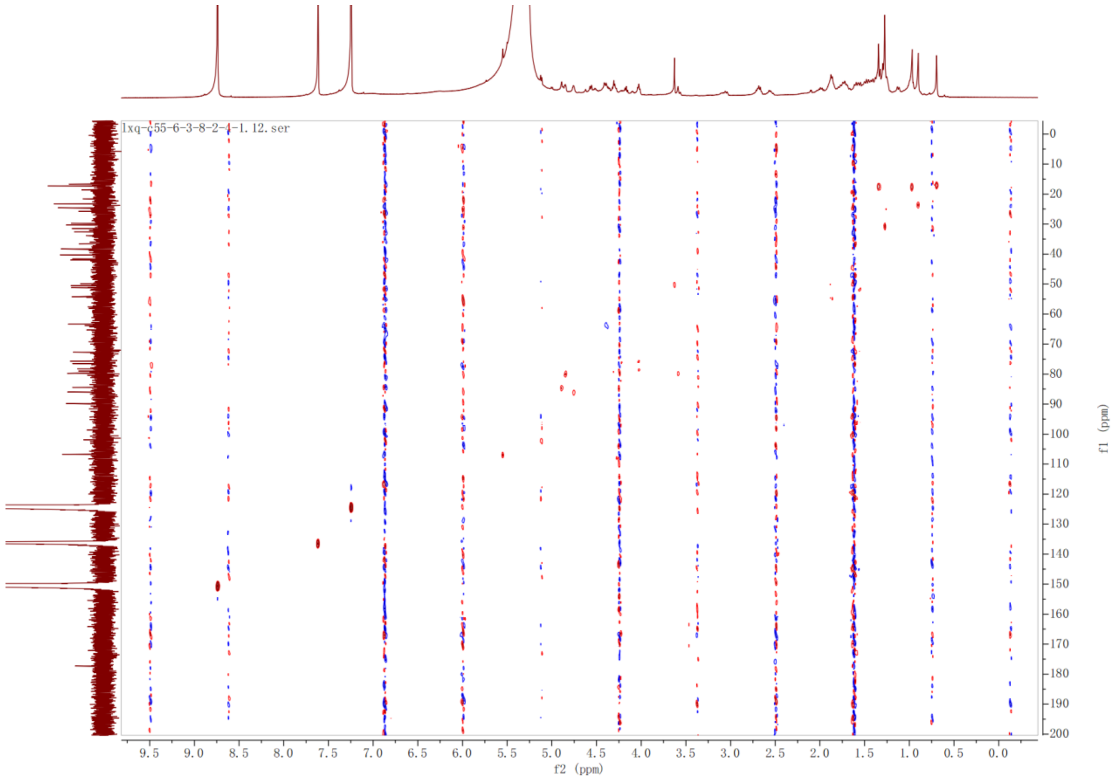


**Figure S21**. HSQC spectrum of compound **3** in pyridine-$d_5$

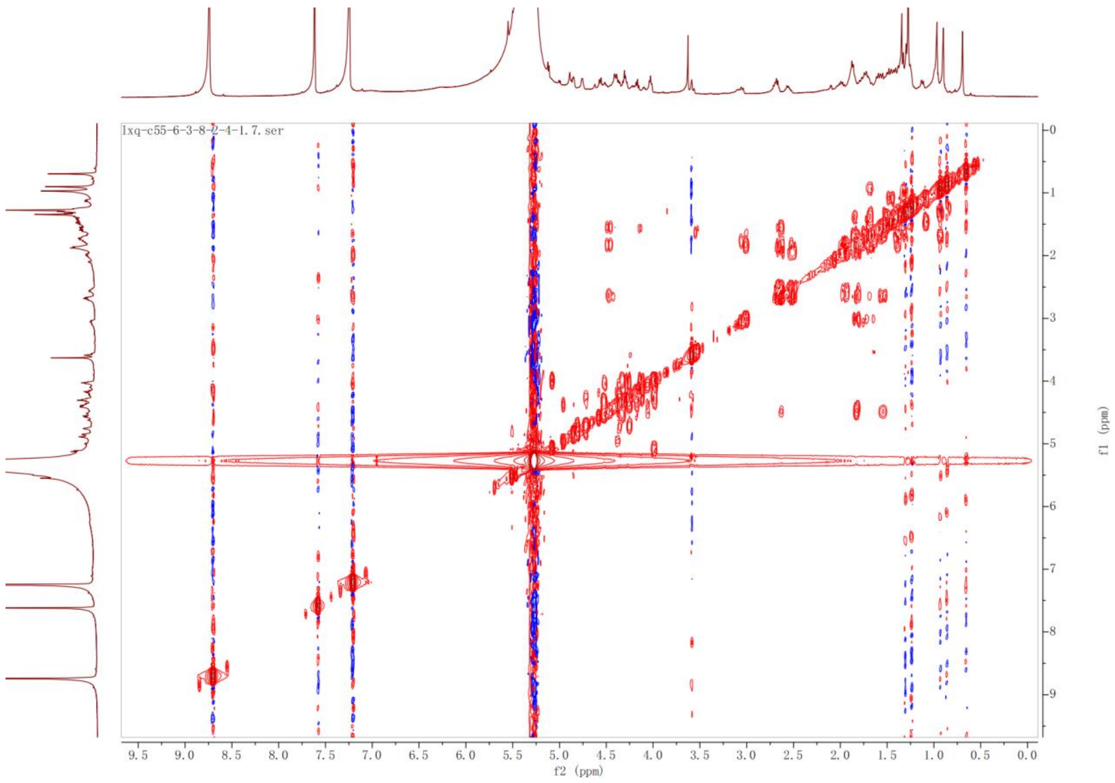


**Figure S22**. $^1$H-$^1$H COSY spectrum of compound **3** in pyridine-$d_5$

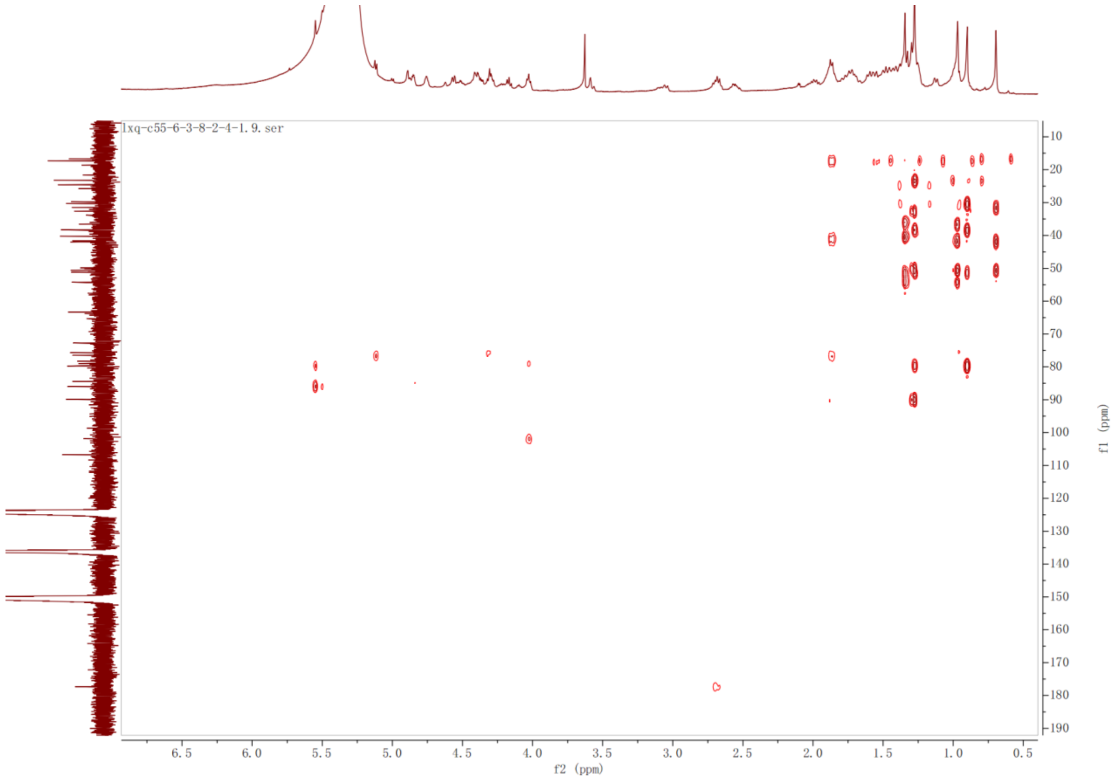


**Figure S23**. HMBC spectrum of compound **3** in pyridine-$d_5$

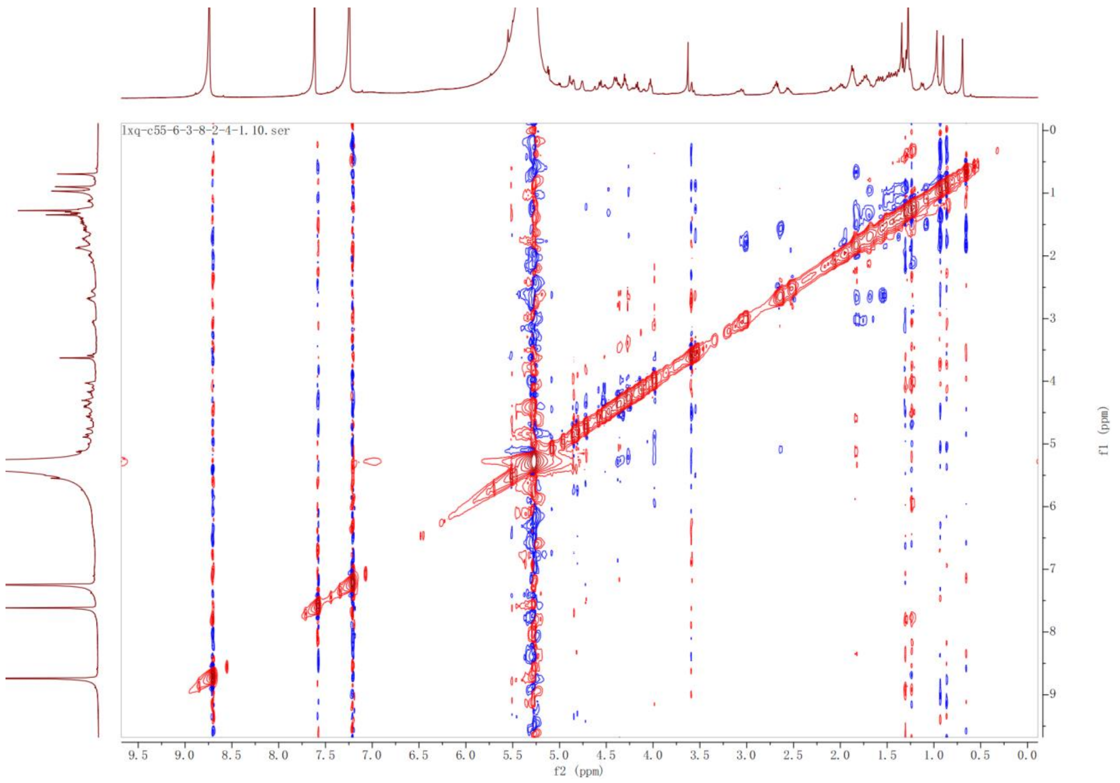


**Figure S24**. ROESY spectrum of compound **3** in pyridine-$d_5$

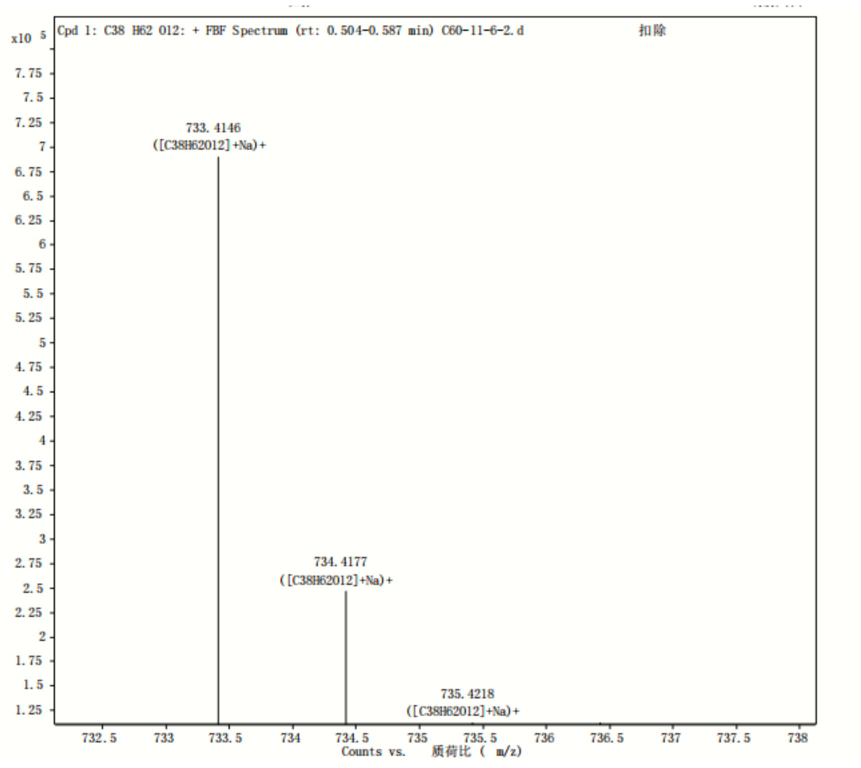


**Figure S25**. HRESIMS spectrum of compound **4**

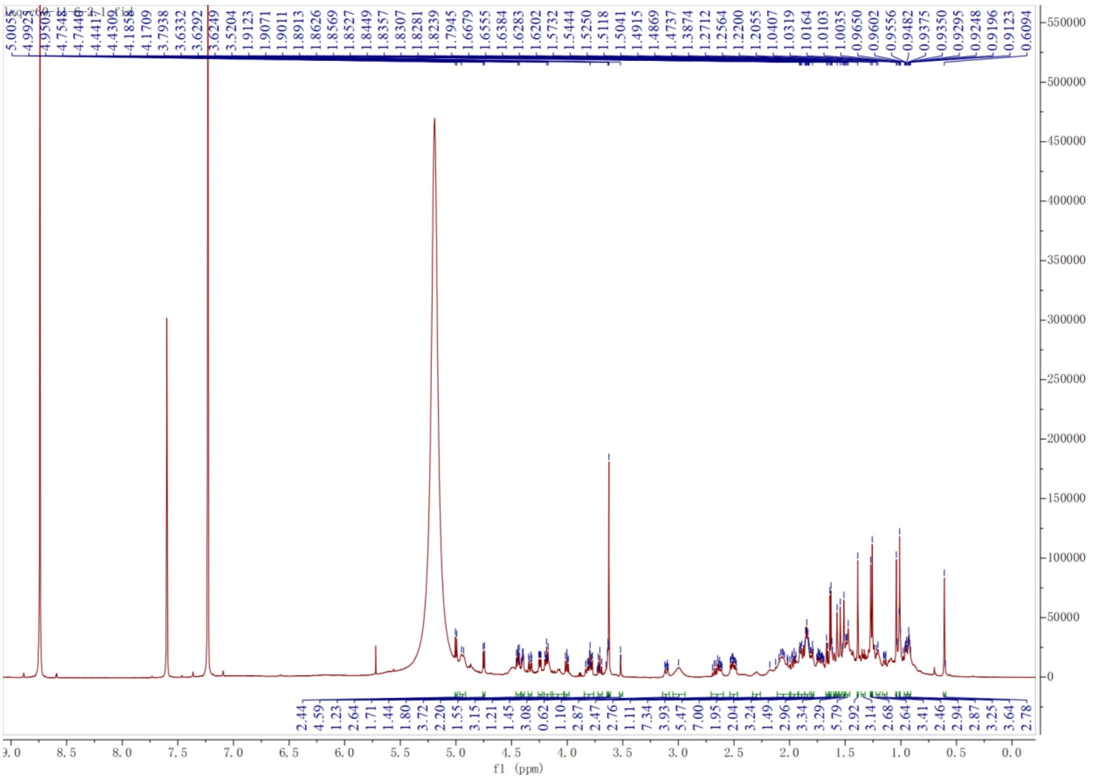


**Figure S26**. $^{1}$H NMR spectrum of compound **4** (600 MHz, pyridine-$d_5$)

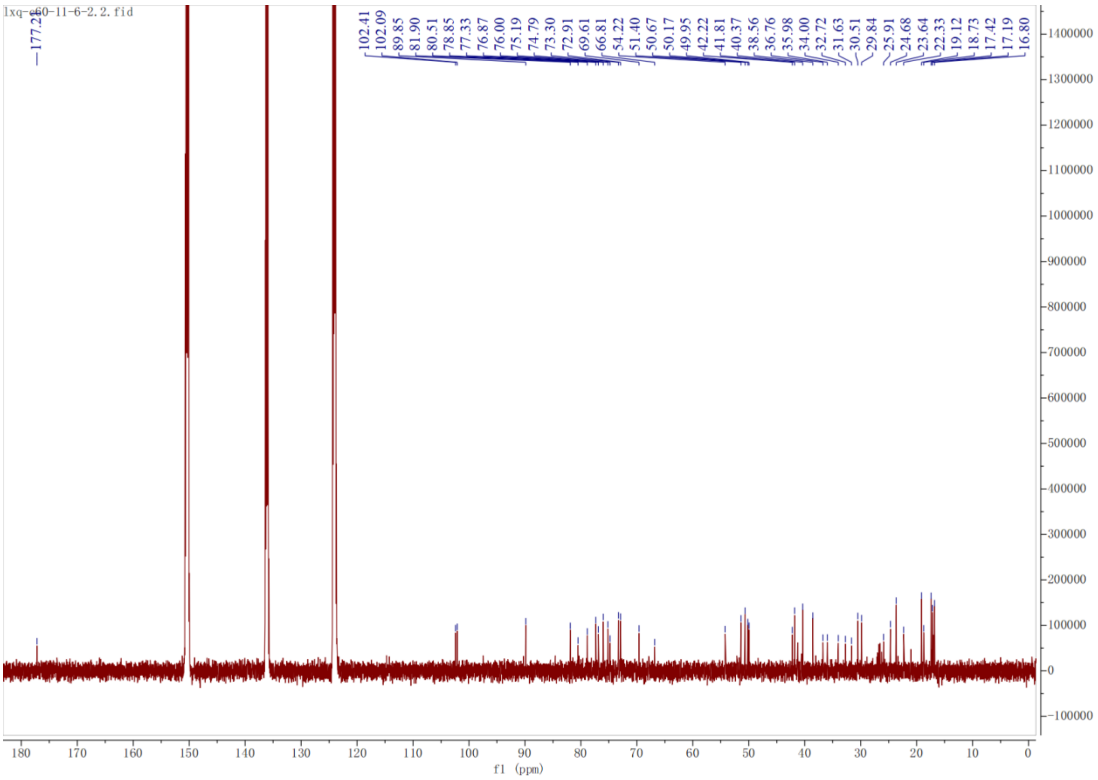


**Figure S27**. $^{13}C$ NMR spectrum of compound **4** (150MHz, pyridine-$d_5$)

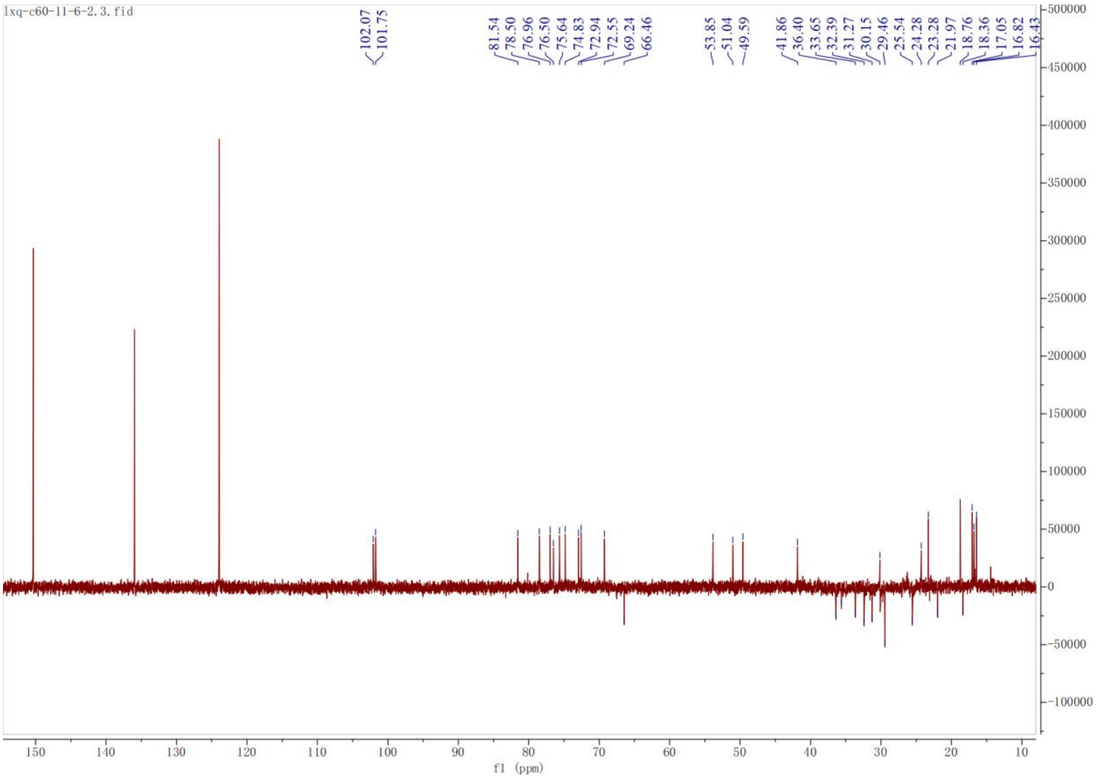


**Figure S28**. DEPT spectrum of compound **4** in pyridine-$d_5$

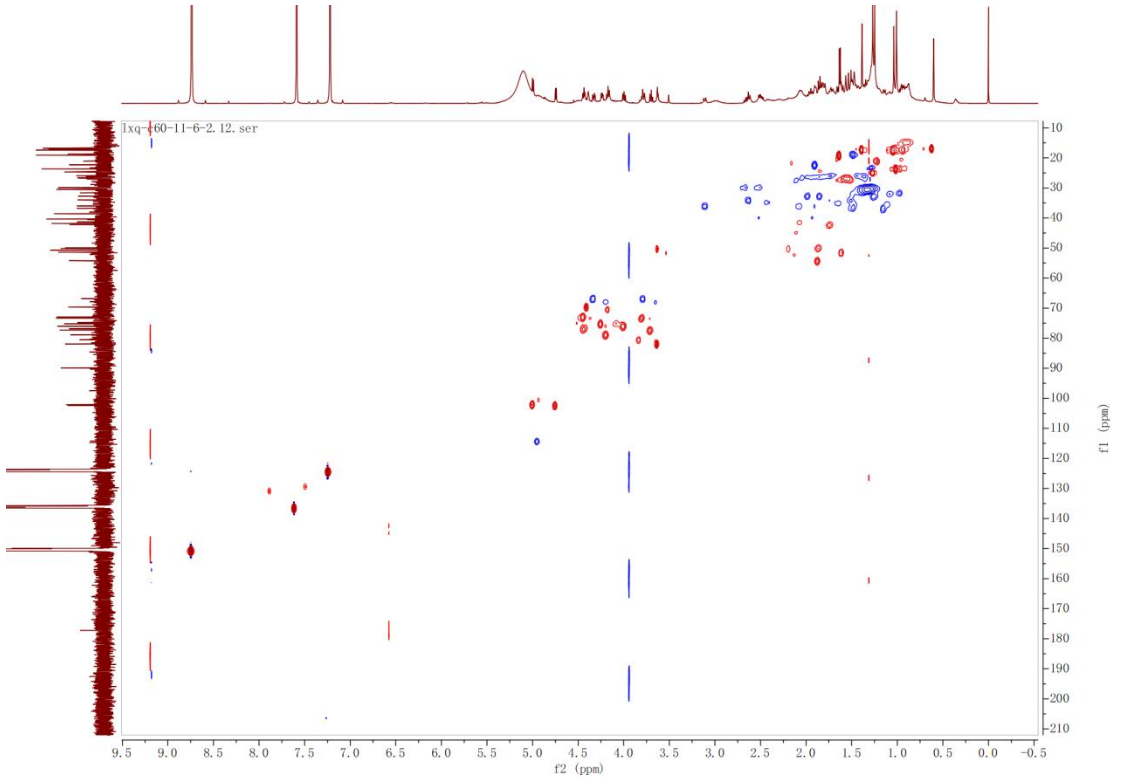


**Figure S29**. HSQC spectrum of compound **4** in pyridine-$d_5$

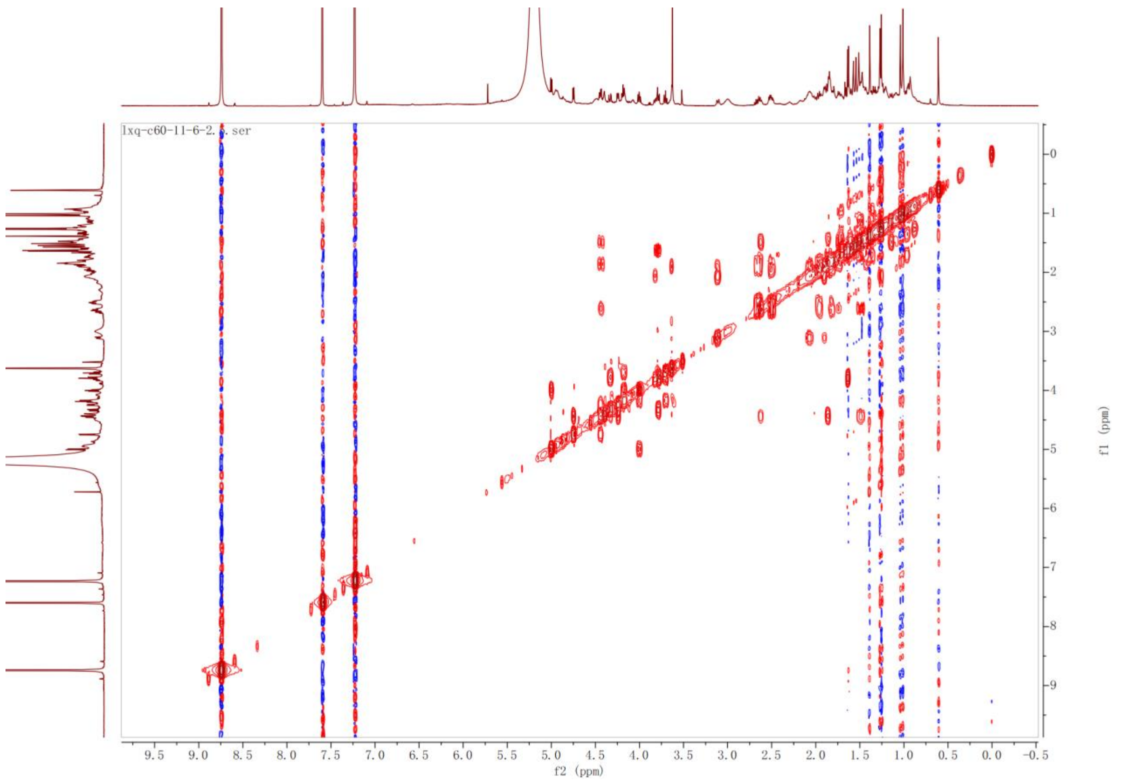


**Figure S30**. $^1$H-$^1$H COSY spectrum of compound **4** in pyridine-$d_5$

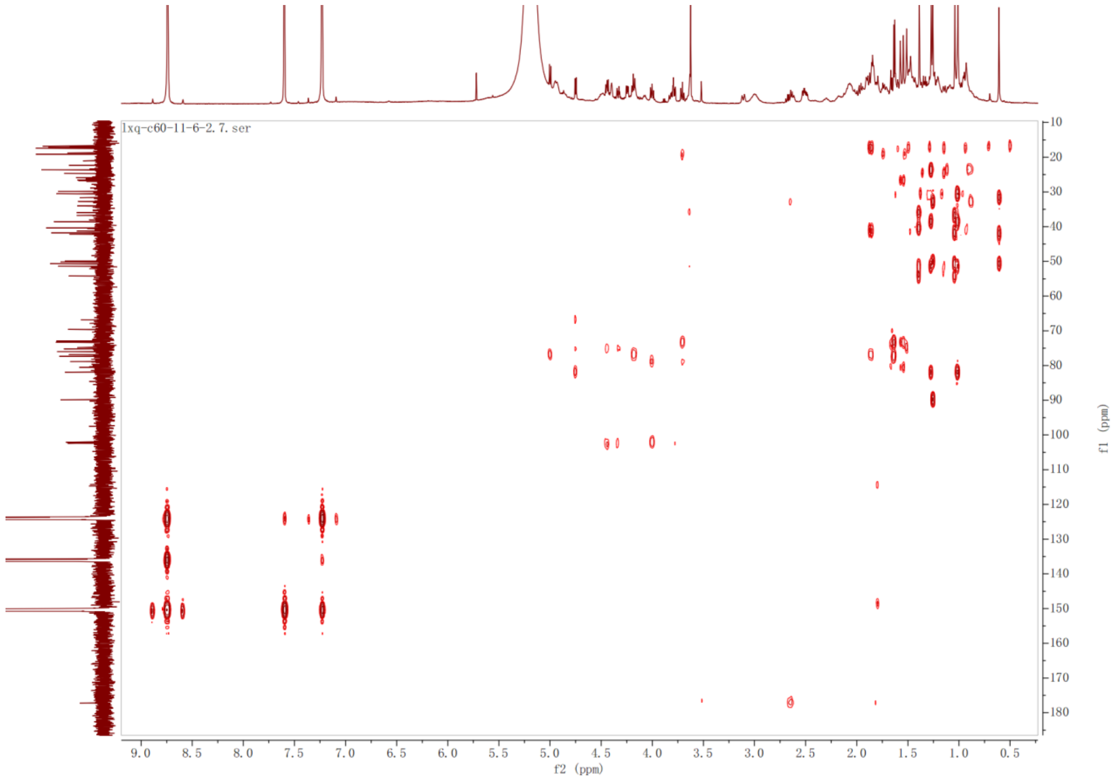


**Figure S31**. HMBC spectrum of compound **4** in pyridine-$d_5$

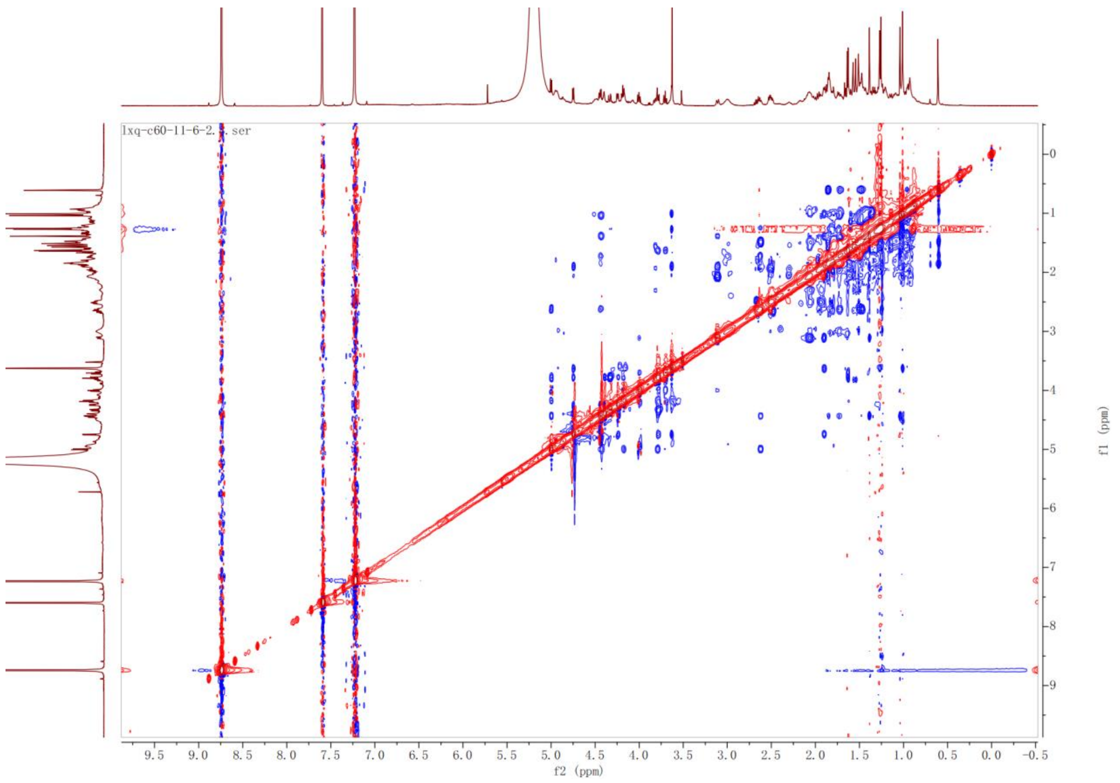


**Figure S32**. ROESY spectrum of compound **4** in pyridine-$d_5$

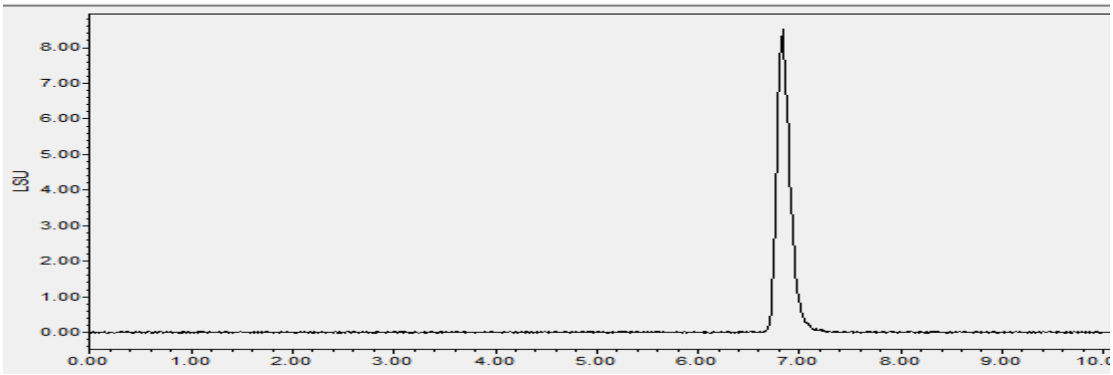


**Figure S33**. The HPLC spectrum of the standard of L-arabinose

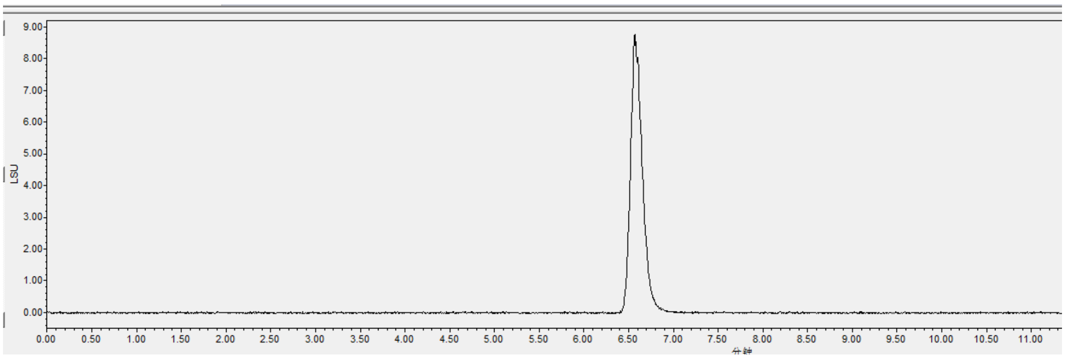


**Figure S34**. The HPLC spectrum of the standard of D-glucopyranose

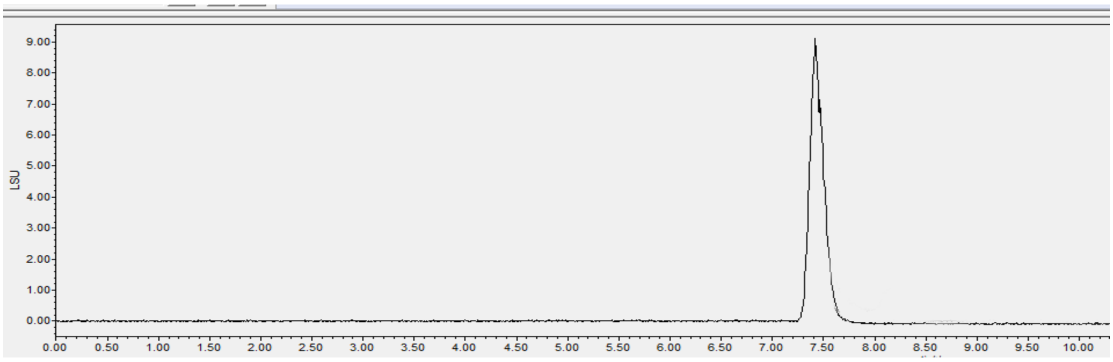


**Figure S35**. The HPLC spectrum of the standard of D-quinovose

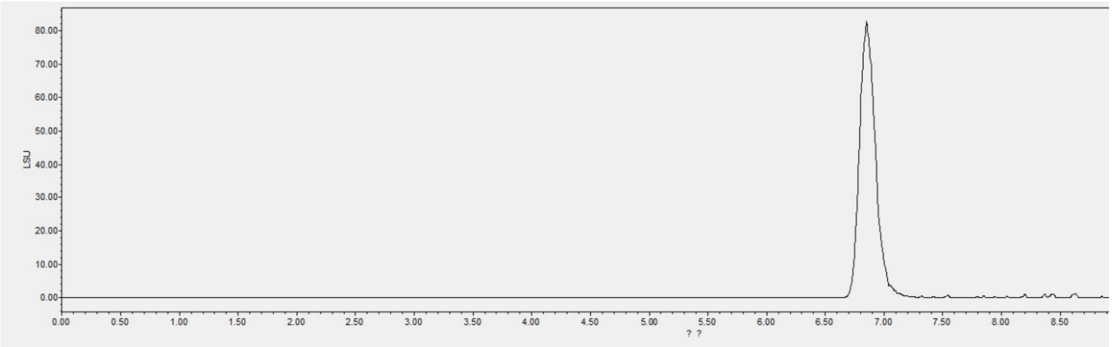


**Figure S36**. The HPLC spectrum of the compound **1** of L-arabinose

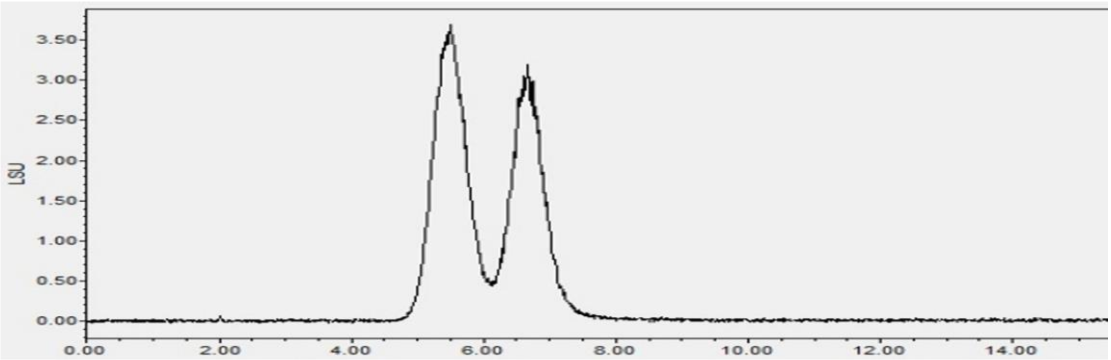


**Figure S37.** The HPLC spectrum of the compound **2** and **3** of D-glucopyranose and L-arabinose

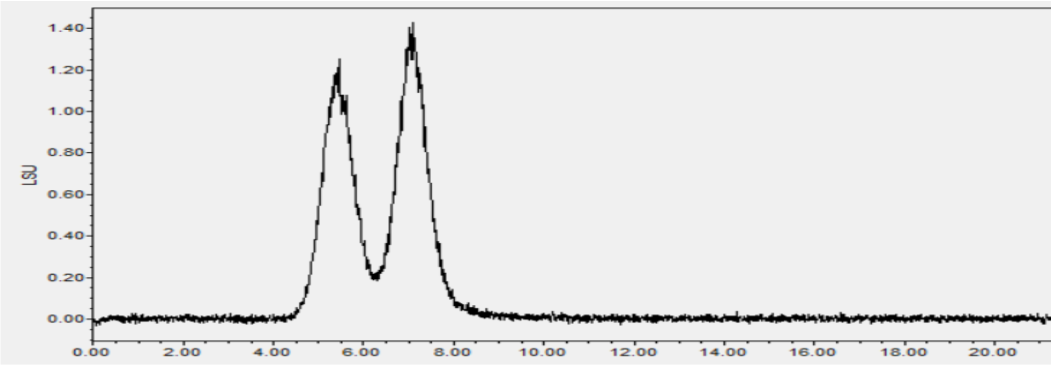


**Figure S38**. The HPLC spectrum of the compound **4** of L-arabinose and D-quinovose
